# Sustained activation of the renin–angiotensin–aldosteron system after fetal exposure to AT1 blockers: Effects on kidney and bone in a preterm newborn

**DOI:** 10.1111/jog.15427

**Published:** 2022-09-13

**Authors:** Gabriele D'Amato, Georgios Rembouskos, Rosa Cafagna, Domenico Dentico, Stefano Palladino, Mariangela Chiarito, Maria Felicia Faienza

**Affiliations:** ^1^ Neonatal Intensive Care Unit “Di Venere” Hospital Bari Italy; ^2^ Fetal Medicine Unit Di Venere and Sarcone Hospitals Bari Italy; ^3^ Radiology Unit Pediatric Hospital Giovanni XXIII Bari Italy; ^4^ Department of Biomedical Sciences and Human Oncology, Pediatric Unit University of Bari “A. Moro” Bari Italy

**Keywords:** AT1 antagonists, bone, fetal exposure, kidney, newborn, preterm, renin–angiotensin–aldosterone system (RAAS)

## Abstract

The renin–angiotensin–aldosterone system (RAAS) plays a key role in development of fetal kidney. Angiotensin‐converting enzyme (ACE) inhibitors or angiotensin II receptor type 1 (AT1) antagonists alter RAAS‐signaling compromising metanephrogenesis, and vascular and tubular development. The result is a fetal “RAS blockage syndrome” that may occur not only following exposure during the second and third trimester, but also after the use of these drugs at the beginning of pregnancy. The in‐utero exposure to AT1 antagonists is not confined exclusively to the risk of neonatal renal failure, but also to skull ossification defect that worsens the neonatal prognosis. We report the case of early arterial hypertension development, marked increase of plasma renin and aldosterone, severe hypocalvaria, and low bone mineralization in a female preterm infant in‐utero exposed to AT1 antagonists.

## Introduction

Hypertension during pregnancy is associated with fetal and maternal morbidities in absence of adequate treatment. Angiotensin‐converting enzyme (ACE) inhibitors and angiotensin II receptor type 1 (AT1) antagonists represent first‐line drugs in primary hypertension for non‐pregnant women. Conversely, this class of drugs is strongly contraindicated during pregnancy, while the therapies of choice are based on methyldopa, beta‐blockers, and calcium channel blockers.^1^ The renin‐angiotensin‐aldosterone system (RAAS) plays a key role in development of fetal kidney.^2^ ACE inhibitors or AT1 antagonists alter RAAS‐signaling impairing metanephrogenesis, and vascular and tubular development. The result is a fetal “RAS blockage syndrome” which may occur not only following exposure during the second and third trimester, but also after the use of these drugs at the beginning of pregnancy.^3^ This syndrome consists of fetal hypotension and anuria with oligohydramnios, leading to Potter sequence, which includes lung hypoplasia, hypocalvaria, limb deformities, growth restriction, and premature birth.^3^ Despite this drug‐related fetopathy, a fair percentage of pregnant women with hypertension still receives AT1 antagonists. Clinical manifestations during neonatal period ranges from minimal morbidity to severe cases requiring intensive care, due to renal damage and cardio‐respiratory failure. In this contest, mortality rate is high and literature data on AT1 antagonist fetotoxicity mainly describe the results of perinatal and neonatal period, while the long‐term outcomes on the recovery of renal failure appear to be limited contrary to those reported with ACE inhibitor fetopathy.^4^ The in‐utero exposure is not confined exclusively to the risk of neonatal renal failure. Indeed, skull ossification defect is a severe side effect that worsens the neonatal prognosis.^5^ This delayed ossification is hypothesized to be due to low‐oxygen tension caused by vasodilation and hypotension.

We report the case of early arterial hypertension development, marked increase of plasma renin and aldosterone, severe hypocalvaria and low bone mineralization in a female preterm infant in‐utero exposed to AT1 antagonists.

## Case Report

**FIGURE 1:**
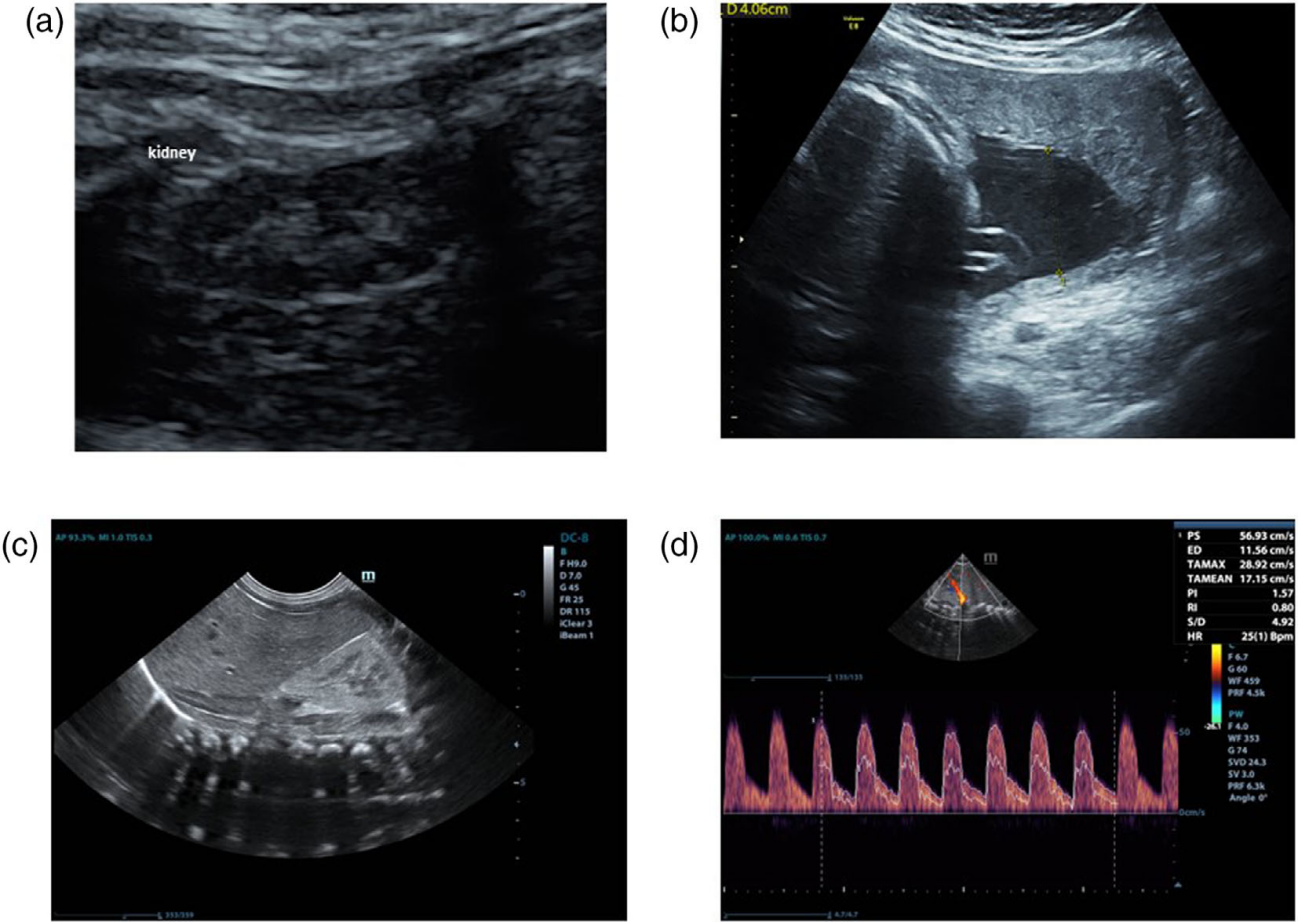
(a) Marked reduction of the amniotic fluid (oligo/anhydramnios) observed at 31 weeks. Normal echogenicity of fetal kidneys. (b) Reappearing of adequate amniotic fluid amount, after maternal therapy suspension, at 32 weeks +5. (c) Ultrasonography of the right neonatal kidney on 3rd day of life showing diffuse increased cortical echogenicity. (d) Mild increase of resistive index in the right renal artery.

We describe the case of a 34‐year‐old pregnant woman with essential hypertension treated with olmesartan medoxomil 40 mg daily, who referred for hospitalization at 31 weeks of gestation for inadequate blood pressure control and severe oligo/anhydramnios (Figure 1a ).

Written informed consent was obtained from the woman and the anonymity was guaranteed.

AT1 antagonist therapy was taken for years before and early in pregnancy, and it was combined with acetylsalicylic acid in the second trimester. During hospitalization, olmesartan medoxomil therapy was suspended and replaced with a calcium channel blocker (nifedipine). After this change, a gradual increase of amniotic fluid and adequate blood pressure control ​​were observed, and the woman was discharged after 10 days (Figure 1b). At 34 weeks of gestational age because of alteration in cardio‐tocographic recording, breech presentation and premature rupture of the membranes, an emergency cesarean section was performed. A female infant was born with normal cardio‐respiratory adaptation. Birth weight: 2.200 g (>25th centile), length: 45 cm (>50th centile), head circumference: 30 cm (>10th centile). A marked reduction of cranial ossification with extensive diastasis of the sutures and fontanelles (hypocalvaria) was observed. On 3rd day of life renal ultrasonography demonstrated diffuse increase in cortical and medullary echogenicity (Figure 1c) with gradient attenuation in both kidneys, and a slight increase in doppler‐derived resistive index (0.80; normal value 0.75 ± 0.06)^6^ in the renal arteries with impaired anterograde diastolic blood flow (Figure 1d). The systolic and diastolic blood pressure values were at 90th centile (85 and 55, respectively) and urinary output was about 2 ml/kg/h with an oral intake of preterm milk formula of 70 ml/kg/day. There was no electrolyte and acid–base imbalance. Glomerular function (microalbumin and serum creatinine), and distal tubular reabsorption capacity (fractional excretion of sodium and phosphorus) were normal, while proximal tubular function was impaired (alpha1‐microglobulin: 18.8 mg/L, normal value: 0–12; beta2‐microglobulin: 3 mg/L, normal value: 0.2). On the 6th day of life blood pressure measurement revealed a full‐blown hypertensive state with mean arterial pressures of 70 mmHg (90/60), corresponding to the 95th centile of neonatal values. Trans‐thoracic echocardiography was normal and pharmacological treatment with amlodipine (calcium channel blocker) at the initial dosage of 0.02 mg/kg as a single administration was started, but due to insufficient blood pressure control it was subsequently doubled. Before starting the antihypertensive therapy, the determination of plasma renin and aldosterone values was carried out, and they appeared markedly elevated (>500 μIU/ml, normal value 4.4–46.1; >100 ng/dl, normal value 2.2–35.3, respectively). At subsequent post‐discharge follow‐up at 2, 4, and 6 months of life, although renal sizes were normal, ultrasound changes (hyperechogenic renal parenchyma and diminished corticomedullary differentiation) persisted (Table 1). Despite hypertension and the persistence of high renin and aldosterone levels and tubular damage, renal function was preserved. Hypocalvaria and contracture of the lower limbs were observed during the follow up. Furthermore, bone mineral density (BMD, g/cm^2^) at the lumbar spine (L1–L4) was measured by Dual‐energy X‐ray absorptiometry (DXA) (Lunar Prodigy Advance) at 2 and 6 months, showing a persistence of low mineralization (0.157 and 0.256, respectively; normal value for age 0.30).^7^

**TABLE 1:** Blood pressure values and biochemical parameters during the follow up

Day of life/months	Blood pressure (mmHg)	alpha1‐microglobulin (mg/L)	beta2‐microglobulin (mg/L)	Renin (IU/ml)	Aldosterone (ng/dl)
3rd day of life	85/55	18.8	3	—	—
6th day of life	90/60	—	—	>500	>100
2 months	88/50	18.5	3	100	58
4 months	87/53	17.9	2.5	71.7	40
6 months	88/55	17.5	2.3	70	35

## Discussion

Appropriate functioning of the RAAS is essential for normal development of the fetal kidney, and mutations of RAAS‐genes or pharmacological blockade of its components promote renal tubular dysgenesis that functionally result in reduced fetal urine production and oligo/anhydramnios.^2^ Mutations in the RAAS‐genes cause severe renal tubular dysplasia (RTD), and affected fetuses usually die before birth or in early neonatal period for anuria, respiratory failure due to lung hypoplasia, or refractory hypotension.^8^ Secondary RTD can be acquired during fetal development due to exposure to various drugs such as ACEi/AT1 blockers, indomethacin, and prostaglandin synthetase inhibitors or in the context of twin–twin transfusion syndrome. Fetal AT1 and angiotensin II receptor type 2 (AT2) receptors are localized to renal differentiating structures in a temporally and spatially specific pattern. AT2 receptors are more predominant in the undifferentiated renal tissue during the first half of pregnancy, while AT1 receptors are expressed in more differentiated tissue in the second half of pregnancy, responsible for final differentiation of nephro‐vascular architecture. Selective blockage of AT1 receptors during second half of pregnancy is therefore associated with severe tubular dysgenesis and renal impairment (Table 2).^5^, ^9^, ^10^, ^11^, ^12^, ^13^

**TABLE 2:** Prenatal and postnatal characteristics of newborns exposed to AT1 antagonists during pregnancy

Author (reference number)	Prenatal ultrasound findings	Gestational age (weeks)	Hypertension	Renal function in first week of life	Renal ultrasound	Hypocalvaria
Schindera et al.^9^	Anhydramnios	35	Yes	Normal	Cortical hyperechogenicity	No
Marchetto et al.^10^	Oligohydramnios	37	No	Impaired	Cortical hyperechogenicity	No
Wegleiter et al.^5^	Oligohydramnios	39	No	Normal	Cortical hyperechogenicity	Yes
Soohyun et al.^11^	Not reported	40	No	Impaired	Cortical hyperechogenicity	Yes
Gang et al.^12^	Oligohydramnios	36	No	Impaired	Cortical hyperechogenicity	No
Petch et al.^13^	Anhydramnios	35	Yes	Impaired	Not determined	No

Accordingly, in our newborn, although fetal exposure to AT1 blockers occurred from the first trimester, the early detection and immediate discontinuation of the drug at the beginning of the third trimester resulted in a normal glomerular function, and a mild proximal tubular dysfunction. However, the possible alteration of nephron‐vascular architecture as documented in fetus exposure to AT1 blockers,^8^ can result in an inappropriate activation of the intrarenal RAAS contributing to the pathogenesis of hypertension and renal damage. This mechanism is confirmed in our patient by the finding of an early hypertension with the persistence of high plasma renin and aldosterone values for several months. Concerning the ossification of the skull, membranous bones have a high degree of vascularity, and a high oxygen tension is required for their growth. Thus, the vasodilation and hypotension due the fetal exposure of AT1 blockers, determine hypoplastic calvaria. However, the concomitant inhibition of osteoblast growth factors involved in bone development has been hypothesized^8^ and could explain the low bone mineralization we found in our newborn. This same theory has also been proposed in cases of hereditary proximal RTD.^14^

### LEARNING POINTS

**•**RAAS‐inhibiting drugs should not be prescribed to pregnant women.

•If such drugs are absolutely indicated, the contraception should be guaranteed for the duration of the prescription.

•Renal damage is related to cell differentiation and to the expression of type 1 receptors in the third trimester of pregnancy.

•It is possible that the RAAS system is involved in the regulation of growth factors that control the development of the enchondral and membranous bones.

## Disclosure Statement

The authors have nothing to disclosure.

## Author Contributions

*Conception and design*: Gabriele D'Amato and Maria Felicia Faienza. *Acquisition of data*: Gabriele D'Amato, Rosa Cafagna and Domenico Dentico. *Analysis and interpretation of data*: Gabriele D'Amato and Mariangela Chiarito. *Drafting of the manuscript*: Gabriele D'Amato. *Technical support*: Georgios Rembouskos and Stefano Palladino. *Critical revision of the manuscript for important intellectual content*: Maria Felicia Faienza.

## Data Availability

The data that support the findings of this study are available on request from the corresponding author. The data are not publicly available due to privacy or ethical restrictions.
